# Efficacy of Platelet-Rich Plasma Intra-articular Injections in Hip and Knee Osteoarthritis

**DOI:** 10.7759/cureus.69656

**Published:** 2024-09-18

**Authors:** Aaisha Shahbaz, Abdulaziz Alzarooni, Vaishnavi Reddy Veeranagari, Kishan Patel, Cara Mohammed, Venkataramana Kuruba, Nirmal Rajkumar, Bakhtawar A Mirza, Momina Rauf, Juan G Maldonado Ramirez, Humza F Siddiqui

**Affiliations:** 1 Trauma and Orthopaedic Surgery, University Hospitals Birmingham, Birmingham, GBR; 2 Medicine, Royal College of Surgeons in Ireland, Dublin, IRL; 3 Medicine and Surgery, Sri Ramachandra Institute of Higher Education and Research, Chennai, IND; 4 Family Medicine, Saba University School of Medicine, The Bottom, NLD; 5 Orthopaedic Surgery, Sangre Grande Hospital, Sangre Grande, TTO; 6 Orthopaedics, All India Institute of Medical Sciences, Mangalagiri, Mangalagiri, IND; 7 Orthopaedics and Trauma, Sri Venkateshwaraa Medical College Hospital and Research Centre, Pondicherry University, Puducherry, IND; 8 Medicine, Shifa Tameer-E-Millat University Shifa College of Medicine, Islamabad, PAK; 9 Internal Medicine, Islamic International Medical College, Islamabad, PAK; 10 Orthopaedic Surgery, Universidad Autónoma de Guadalajara School of Medicine, Mayaguez, PRI; 11 Internal Medicine, Jinnah Sindh Medical University, Karachi, PAK

**Keywords:** hyaluronic acid, intra-articular injection (iai), leukocytes, osteoarthritis, platelet rich plasma (prp)

## Abstract

Osteoarthritis (OA) is a chronic degenerative disorder that causes significant pain and functional limitations. Platelet-rich plasma (PRP) therapy has gained considerable attention in recent years in the treatment of musculoskeletal injuries. In this narrative review, we aim to investigate the role of intra-articular PRP injections in the treatment of knee and hip OA. The review also discusses different classifications of PRP based on composition. Furthermore, this narrative review also identified various limitations of PRP therapy in OA. PRP is classified into different types based on cell content and fibrin architecture, including pure platelet-rich plasma (P-PRP), leukocyte- and platelet-rich plasma (L-PRP), pure platelet-rich fibrin (P-PRF), and leukocyte- and platelet-rich fibrin (L-PRF). Various clinical trials have shown that PRP is an effective option for the treatment of knee and hip OA. However, the superiority of PRP over hyaluronic acid has been reported inconsistently. This variability can be attributed to PRP preparation techniques. The safety profiles of PRP are generally favorable and the adverse effects are generally mild in nature. Although there is sufficient evidence in support of PRP in the treatment of OA, the long-term effects of PRP have not been reported. Further studies should focus on longer follow-up periods to identify the efficacy and safety of PRP in treating knee OA. There is also a need for standardization of PRP preparations in OA management.

## Introduction and background

Osteoarthritis (OA) is a chronic degenerative disorder that poses significant public health concern globally with a burden of 7.6% [1]. The most commonly affected region is the knee with an estimated global prevalence of 22.9% in individuals above the age of 40 years [2]. Cartilage degradation is the central feature of OA. This is primarily because articular cartilage is anatomically positioned at the forefront, absorbing and distributing mechanical loads applied to the articular joint, and providing a low-friction surface to facilitate mobility [3]. Initially, OA was considered only to affect articular cartilage; however, recent research has shown that it affects all types of cartilage [4]. OA affects all the tissues within and surrounding the joint. These effects primarily include osteophyte formation, synovial inflammation, ligament degeneration, articular cartilage degradation, and thickened subchondral bone [5]. OA is associated with a significant decline in quality of life attributable to pain, discomfort, and impairment of function due to the deterioration of the joint. The main treatment approaches for OA primarily focus on pain management. Current guidelines suggest the use of both drugs, including oral non-steroidal anti-inflammatory drugs (NSAIDs), analgesics, symptomatic slow-action drugs for OA (SYSADOA), and non-drug treatments like physical therapy for the management of this condition [6].

Treatments targeted at managing pain provide short-term benefits. Currently, there are no medical treatments that slow down progressive degeneration. Platelet-rich plasma (PRP) has emerged as a novel option in OA treatment as it has high concentrations of growth factors. PRP components have the ability to influence the pathogenic processes of OA [7]. However, there is a paucity of conclusive evidence concerning its standard dosage, preparation techniques, and efficacy level [8]. The use of PRP in tendinopathy, soft tissue injuries, ligament tear, chondropathy, and healing of bone damage has been documented in the literature; however, there is a lack of significant evidence to support their use in these conditions. The efficacy of PRP has also been shown in the recovery of musculoskeletal lesions, with minimum risk of immune reactions and transmission of contagious diseases [9]. PRP is an autologous product that is enriched with high platelet counts. The form of PRP depends entirely on its constituents. Each PRP composition varies with respect to the presence of leukocytes, platelets, and additives. Additives such as calcium chloride and thrombin are used to ensure the regenerative potential of PRP by stimulating platelet activation and clotting and enhancing proliferative capacity [10]. The mechanism of action of PRP involves platelet activation and granulation, the release of growth factors such as platelet-derived growth factors (PDGF), vascular endothelial growth factor (VEGF), Type I insulin-like growth factor (IGF-I) and transforming growth factor (TGF)-β [11]. This leads to the onset of angiogenesis and the stimulation of stem cells [12].

Despite the positive effectiveness of PRP being reported in several studies, the debate persists regarding the clinical efficacy of PRP. The purpose of this narrative review is to fill this gap in the literature. In this review, we summarize the current evidence on the role of intra-articular (IA) injections of PRP in treating hip and knee OA. The objective of the review is to investigate the superiority of PRP treatment over conventional treatments, understand the appropriate techniques and standard dosage of PRP, and the safety of PRP injections.

## Review

PRP preparation

Platelet-rich plasma (PRP) is produced using autologous blood, which is blood obtained from the patients themselves. In the disciplines of orthopedics and sports medicine, the use of tissue regeneration and healing has gained popularity [13]. Centrifugation is used to segregate the distinct constituents of blood and enhance the abundance of platelets in PRP, which is generated by obtaining a blood sample from a patient and subjecting it to further processing. PRP produced utilizing different commercial methods and techniques may exhibit variations in platelet concentration, white blood cell composition, and fibrin structure. The differences have an effect on the biological activity and efficacy of PRP as a treatment [14].

**Figure 1:**
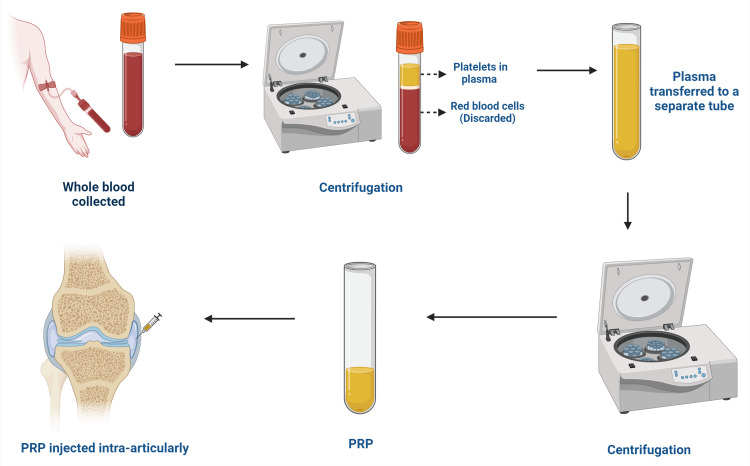
Overview of PRP preparation PRP: platelet-rich plasma This figure has been created by the authors using Biorender.

In order to concentrate platelets and their growth factors in a small amount of plasma during PRP preparation, it is necessary to minimize the concentration of red and white blood cells [15]. Typically, a blood sample of 10-60 mL is taken from patients and processed using either a two-spin or one-spin centrifugation method [16]. The two-spin procedure entails first centrifuging platelets at a high velocity, and then subsequently separating red blood cells from the PRP at a lower velocity [17]. The platelets are collected from the buffy coat layer using the one-spin method, which involves a single rotation at a reduced speed [18]. There are two methods for preparing PRP: open and closed method. In the open technique, the blood is exposed to the conditions of the laboratory during the preparation process, whereas in the closed technique, it is not exposed due to the utilization of commercial kits [19]. The resulting PRP may be L-PRP or P-PRP, depending on the white blood cell content [20] (Figure 1).

Presently, the standardized preparation procedures are inadequate. Processing quantitative standards (PQSs) account for production factors including time, volume, and pressure. Aspects such as the quantity of blood processed, length of time, temperature, and rate of centrifugation and incubation are taken into consideration. Processing qualitative standards (PQLSs) ensures the quality of the substance and procedure used. PQLSs ensure the use of appropriate calcium chloride (CaCl2), equipment of feasible weight and size, and process completion within 30 minutes in uncontaminated surroundings is maintained. As novel critical quality attributes (CQAs) are being employed in the preparation of PRP in regard to purity and potency, standardized benchmarks can be set for PQSs and PQLs, leading to the production of high-quality, effective, and safe PRP [21].

Formulations of PRP

PRP can be formulated in various ways with different properties and varied efficacies to enhance the therapeutic potential of a wide spectrum of clinical applications. The formulation of PRP depends on factors such as the centrifugation protocol, activation status, and addition of adjuncts. The myriad properties of PRP have varied levels of efficacy for different diseases, which are vital to consider in clinical practice and research studies [13].

Ehrenfest et al. described four categories of PRP based on the presence of leukocytes and fibrin structure. Pure PRP (P-PRP), also known as leukocyte-poor PRP, and leukocyte-rich PRP (L-PRP) both contain low-density fibrin and are differentiated based on the absence and presence of leukocytes, respectively. These products can only be applied as gels and are not available in injectable form. Platelet-rich fibrin (PRF) or second-generation PRP products are formulations that constitute high-density fibrin with or without leukocytes [16].

Inactive PRP: Liquid PRP and PRGF

Concentrated platelets that have not been activated outside of the body with plasma constitute liquid PRP. Platelets and their growth factors are present in substantial amounts in the material, and they may be released when the body activates them at a particular tissue location [13]. An altered form of PRP known as plasma rich in growth factors (PRGF) is created without the requirement for thrombin as an activator by adjusting its pH [15].

Both liquid formulations of PRP and PRGF are often used for skin treatments, IA injections, and other situations where liquids are required. Some of the benefits of this substance are its ease of administration, quick preparation time, minimal risk of allergic response and inflammation, and capacity to gradually release growth factors. When comparing liquid PRP and PRGF to activated PRP formulations, it is possible that the latter may have a lower effectiveness in terms of releasing and absorbing growth factors [13, 15].

Activated PRP

Platelets must be activated to encourage tissue regeneration and repair. A thrombin and calcium chloride solution combined with PRP results in activated PRP, which is a viscous substance. This drives the platelets to release growth factors resulting in the formation of a fibrin clot. Four vital techniques can be engaged for platelet activation, which necessitates the use of autologous thrombin, CaCl2, 10% collagen type I, and a mixture of CaCl2 and thrombin. Platelet activation is necessary for the release of numerous growth factors and cytokines during application. Some studies showed that variable platelet activators impact the cytokine release process, such as collagen leads to persistent release, while thrombin can initiate an immediate release. Another study revealed that transforming growth factor (TGF)-β, fibroblast growth factor (FGF), and platelet-derived growth factor (PDGF) release was significantly steep within the first 48 hours of application and then gradually declined subsequently [13, 22].

The activated PRP can be used as a surgical adhesive or implant to deliver growth factors at the tissue site. The advantages of activated PRP include its ability to provide a localized and sustained release of growth factors, enhance tissue repair and regeneration, and serve as a scaffold for cell attachment and proliferation. However, the use of thrombin as an activator may have potential drawbacks such as inducing an immune response, promoting inflammation, and inhibiting bone morphogenetic protein-2 activity [23].

L-PRP vs. P-PRP

PRP can be classified as L-PRP or P-PRP depending on the white blood cell content. L-PRP, containing a high concentration of leukocytes, can release pro-inflammatory cytokines and proteases into the tissues when used clinically and may inhibit tissue healing. P-PRP is predominantly associated with anabolic and anti-inflammatory properties and a decreased leukocyte count. The optimum selection of P-PRP or L

-PRP is based on the patient's specific therapeutic indication and the equilibrium between their pro-inflammatory and anti-inflammatory effects. Additional research is needed to determine the optimal amount of white blood cells in PRP for different therapeutic uses [14, 24].

Regulations

PRP regulations differ throughout nations worldwide. The Food and Drug Administration (FDA) in the United States is responsible for regulating PRP, which falls under the category of human cells, tissues, and cellular and tissue-based products (HCT/Ps) [25]. The Public Health Service (PHS) Act's section 361 addresses cellular and tissue products intended for homologous use that are subject to little modification [26]. The FDA has established special regulatory pathways for these products. For PRP products that are non-homologous or undergo more than minimal modification, the FDA has even recommended stricter regulations. More accurately, PRP products are being promoted by companies for non-homologous uses, including treating diseases unrelated to the injected tissue, for which the FDA has shown concern and issued many warning letters [27]. Additional recommendations from the FDA have been made to regulate PRP products under PHS Act section 351. It is required under this act to submit premarket approval applications confirming the efficacy and safety of PRP via clinical trials [28].

Known as an advanced therapeutic medical product (ATMP), PRP is governed in Europe by the European Medicines Agency (EMA) [29]. Under EMA, it is mandatory to prove via clinical trials that the efficacy of pharmaceutical drugs outweighs the side effects for the treatment of specific conditions under consideration to acquire permission to market the drug. EMA mandates that the same rule is applicable to PRP. Many European countries classify PRP as a “non-standardized medicinal product”, for which good manufacturing practices (GMP) standards must be accomplished. Switzerland has necessitated similar standards and practices in the Therapeutic Products Act (TPA) [30]. In the European Union, PRP is regulated under Directive 2001/83/EC of the European Parliament and of the Council of 6 November 2001 on the Community code regarding medicinal products for human use. In Spain, the Spanish Agency of Medicines and Health Care Products mandates that only a medical physician, dentist, or podiatrist can administer PRP, assuming responsibility for the quality, efficacy, and safety of the treatment. It was a landmark report as it categorized PRP as a human-used therapeutic modality rather than blood procured product, resulting in stringent regulations [31].

Mechanisms of PRP action

Platelets and plasma are both naturally occurring products within human blood. PRP has a higher concentration of platelets, two to eight times more than its natural proportions in the body. This is achieved by multiple rounds of centrifuging. The primary functions of platelets are well-established as homeostasis and thrombosis. They also play an integral role in many steps of leukocyte recruitment which is essential to host defense and immune response [32]. Recent studies have shown that activated platelets release a combination of cytokines, growth factors (GF), and extracellular matrix modifiers which are vital to healthy wound healing [33].

PRP contains adhesive proteins - fibrin, fibronectin, and vitronectin - which enable the formation of a fibrin gel that provides a mesh-like scaffolding to further improve healing [34]. Upon injection, PRP acts at the target site by stimulating the main processes of migration, proliferation, cell differentiation, and angiogenesis [35]. This is facilitated via growth factors including platelet-derived growth factor (PDGF), vascular endothelial growth factor (VEGF), epidermal growth factor (EGF), and transforming growth factor-β (TGF-β) [36].

When angiogenesis begins, VEGF binds to VEGF receptor 2 which activates endothelial cells. These endothelial cells become tip cells and then develop into endothelial stalk cells, ultimately proliferating to establish a stable vessel lumen. Basic fibroblast growth factor (bFGF) is another vital element involved in the initiation of angiogenesis. Similar to VEGF, it increases proliferation and extends the survival of the endothelial cells [37]. Endothelial cell proliferation is further promoted by PDGF, which also contributes to the chemotaxis of fibroblasts, chondrocytes, and mesenchymal stem cells (MSC). TGF-β boosts undifferentiated mesenchymal cell proliferation, while also increasing chondrogenesis [34]. The latter stages of angiogenesis prioritize the stabilization of the newly formed lumen of the vessel. VEGF-led neovascularization often results in leaking unstable vessels. Angiogenic factor Ang1, released from thrombin-activated platelets, facilitates vascular maturation by increasing stability and decreasing leakage. Platelet-derived growth factor-BB (PDGF-BB) contributes to maturation by assisting in the recruitment of pericytes which stabilize via proliferation and migration to the emerging vessel [37]. Finally, it is important to note the value of inhibitory and regulatory signals in angiogenesis arising from platelets. An uninhibited stimulation of proangiogenic factors could result in tumor angiogenesis and an abnormally excessive growth of vascular structures. This often leads to the formation of immature and unstable blood vessels. To prevent this, platelets host a wide variety of inhibitors including thrombospondin-1 (TSP-1), endostatin, and platelet factor 4 (PF4). These factors impede endothelial cell proliferation by preventing VEGF from interacting with endothelial cell receptors and thus are vital for maintaining the balance [37, 38].

OA is an inflammatory process in which constant mechanical stress leads chondrocytes to release inflammatory cytokines, such as IL-1B, TNF-a, and IL-8, which in turn recruit leukocytes to the site of injury via chemotaxis. Without treatment for the inflammation caused by these cytokines, these leukocytes destroy the joint space and worsen the pain felt by the patient [39]. When PRP is introduced to the site of injury, platelets begin to degranulate. They release inflammatory cytokines such as CXCL7, which can contribute to neutrophil chemotaxis by binding to their chemokine receptors. The chemokine can be inhibited by the plasma proteins found in PRP reducing inflammation. Activated platelets can also release CD-40L which binds to CD-40 on various other cells creating inflammatory effects. PRP also releases anti-inflammatory proteins such as IL-1RN, which blocks IL-1B and reduces joint breakdown. Monocyte recruitment and differentiation are imperative to resolving the inflammation caused by OA. Platelet factor 4 (CXCL4) plays an important role in ensuring the survival and differentiation of monocytes. The protein pushes the monocytes down a pathway of differentiation to eventually become suppressor cells, which are also helpful in reducing inflammation. Additionally, platelets also release antimicrobial proteins that prevent pathogens from occupying the joint space [38].

Efficacy of PRP in OA

Hip OA

Multiple randomized control trials (RCTs) are being conducted to confirm the therapeutic advantages of PRP injections in the osteoarthritic hip joints to control pain and improve functionality. Amongst these, Nouri et al. conducted a randomized control trial in Iran, which involved 105 patients being randomly allocated to three parallel groups i.e. hyaluronic acid (HA), PRP, and PRP + HA. These patients received two injections in Grade 2 and 3 osteoarthritic hip joints at two-week intervals. Assessments were made before the injections and at two- and six-month intervals after the injection. The results showed that the Visual Analogue Scale (VAS), Western Ontario and McMaster Universities Osteoarthritis Index (WOMAC), Lequesne total scores and activities of daily living (ADL) showed significant improvement in PRP and PRP + HA groups at six months, indicating that PRP and PRP+HA have far superior therapeutic effects at six months post-injection [40]. In another study, Doria et al. analyzed the efficacy of PRP as opposed to HA injections for addressing early hip OA in a controlled clinical trial. PRP exhibited subjective clinical improvement without demonstrating a significant superiority over HA among middle-aged individuals experiencing moderate OA symptoms. Both interventions resulted in similar improvements in clinical evaluations, utilizing the WOMAC subscale and Harris Hip Score (HHS), throughout the six- and 12-month follow-up periods. The analysis determined that PRP, despite its safety, might not be the most suitable primary treatment choice for this specific patient population [41].

Similarly, Dallari et al. led a randomized controlled trial to compare the effectiveness of different IA injections for hip OA in patients aged 18 to 65. The study assessed the effects of giving injections of L-PRP, a combination of L-PRP and HA, or only HA every three weeks to the affected patients. The findings showed that those who received L-PRP injections had the least pain at all follow-up appointments. Their pain levels and their joint function and stiffness, assessed by VAS and WOMAC scores had shown improvement at two months and six months compared to the other treatment groups. A positive correlation was also identified between interleukin-10 levels in PRP and pain reduction. The study concluded that L-PRP injections significantly improved clinical outcomes in hip OA patients, proving more effective than HA alone or combined with HA [42]. In another similar RCT involving 43 patients, PRP significantly reduced visual analog scale (VAS) pain scores at four weeks, with 85% of patients experiencing at least a 30% reduction in pain. However, these benefits were not maintained at 16 weeks [43]. Another study reported significant improvement in pain and function at one and three months, with 70% of patients showing favorable outcomes, but no significant difference between PRP and hyaluronic acid (HA) at 12 months [44].

In one of the recent meta-analyses, Lim et al. compared the effects of single-dose PRP versus multiple PRP injections, low-dose (<15mL) versus high-dose (≥15 mL) PRP and LP-PRP versus LR-PRP. The results showed that there was a reduction of pain with PRP injections, however, a significant reduction in VAS score was seen only at one to two months follow-up. The evidence collected in this analysis suggested that a larger reduction of pain is achieved with single, low-dose, and LP-PRP injections [45]. Another meta-analysis comprising 334 participants showed that PRP treatment was found to be more effective in earlier stages, with longer-term evaluations from four to 12 months yielding diverse results [46]. A meta-analysis including seven RCTs with a total of 478 patients, conducted to evaluate the efficacy of PRP versus HA showed that WOMAC and VAS pain scores were significantly reduced in the PRP group at six months post-injection, whereas both PRP and HA groups had similar standard mean WOMAC and VAS scores at one to two and twelve months. However, there was no statistically significant difference noted in HHS for both groups at six and 12 months [47].

These findings contradict another systematic review and meta-analysis of level I and level II RCTs conducted by Belk et al. They included six RCTs with 211 patients undergoing PRP IA injections and 197 patients who received HA injections. They found that WOMAC, VAS, and HHS scores did not have any statistically significant difference between the two groups. Therefore, patients who receive either of the two interventions can be expected to experience short-term benefits [48]. Similarly, Garcia et al. found that PRP could enhance outcomes and alleviate pain for up to a year in cases of hip OA. However, these improvements in pain at two, six, and 12 months showed no statistically significant differences between PRP and hyaluronic acid treatments [49]. Furthermore, a systematic review led by Berney et al. analyzed five trials involving 185 patients with hip OA who received PRP injections, with follow-ups at six and 12 months. The study aimed to compare PRP with hyaluronic acid injections, focusing on patient outcomes measured by the WOMAC and VAS scores. Though PRP showed improvement in patient outcome scores at six and 12 months, the results were not significantly different from those treated with HA alone [50].

A study conducted by Gazendam et al. analyzed 11 randomized controlled trials involving 1,353 patients indicating that treatments like corticosteroid (CS), HA, and PRP failed to demonstrate a significant enhancement in pain or function scores compared to saline injections during the initial two to six months. However, at six months, PRP had the highest probability of being the most effective treatment option, with a surface under the cumulative ranking curve (SUCRA) value of 71.5%. While all interventions except HA+PRP showed clinically important improvements, PRP did not demonstrate superior efficacy over placebo or other treatments. Consequently, the study concludes that PRP is not strongly supported over other injectable options for managing hip OA [51]. Contrarily, Zhao et al. identified that PRP was the leading treatment for sustained pain relief, maintaining its efficacy for up to six months. The study incorporated data from 11 randomized controlled trials encompassing 1,060 patients, out of which four RCTs specifically included PRP injections, with 157 patients receiving PRP alone. The meta-analysis revealed that PRP had the lowest SUCRA value for VAS scores at six months, highlighting its effectiveness in offering extended pain relief. The findings from this study suggest that when PRP was a promising intervention for managing hip OA pain, outperforming comparisons with HA, corticosteroids, and HA combined with PRP [52]. Another meta-analysis reported that while PRP was effective in reducing pain at two months, it did not provide significantly improved HHS and WOMAC scores at 12 months compared to HA [53]. A study involving 1176 patients treated with HA, PRP, and other treatments found that PRP was associated with significant pain reduction at two months but no notable difference at six or 12 months [54].

Knee OA

An RCT conducted by Raeissadat et al. investigated the efficacy of PRP in knee OA. Their findings showed that there was a significant improvement in mean WOMAC and VAS scores after treatment (p<0.05). Furthermore, radiologic variables such as patellofemoral cartilage volume, synovitis, and medial and lateral meniscal disintegrity also showed significant improvement in the PRP group (p<0.05) [55]. Similarly, in another RCT, Elik et al. showed that at the six-month mark, all WOMAC score parameters were also lower in the PRP group compared to the placebo group (p < 0.05) [56]. Superior results have been reported by Partan et al. for PRP in knee OA compared to hyaluronic acid. They further revealed that PRP treatment was associated with an increased level of platelet-derived growth factor (PDGF)-BB which can explain the growth and regeneration of PRP [57].

PRP has been compared to several other injectables in various studies. Bennell et al. found no significant difference compared to placebo, indicating variability in outcomes [58]. Park et al. performed a clinical trial comparing PRP with HA. A single injection of L-PRP and HA was administered, and International Knee Documentation Committee (IKDC) scores were better among the PRP group as compared to HA. Authors also concluded that growth factor concentration in PRP should also be accounted for when administered [59]. Di Martino et al. studied the differences in efficacy between L-PRP and P-PRP among patients with knee OA. Every patient received three weekly injections and reported clinical improvement at follow-up after 12 months from both groups. However, no statistical difference was found between the outcomes of the two modalities [60]. Another study reported marked improvement in functional status and reduction in clinical symptoms after administration of PRP and HA at 24 months follow-up. PRP did not exceed in outcomes compared to HA, although the frequency of re-administration was significantly lower [61]. Huang et al. conducted an RCT to compare outcomes of different HA formulations in combination with PRP and concluded that both treatment regimens equally benefitted the patients [62]. Pishgahi et al. conducted an RCT using novel IA biological agents including PRP, autologous conditioned serum (ACS), and dextrose prolotherapy. Substantial reduction in pain and enhancement in knee function was reported among patients who received PRP and ACS, with superior results in ACS groups. However, no improvement was observed among patients treated with dextrose prolotherapy [63]. Forogh et al. reported that a single IA injection of PRP proved more effective in mitigating pain and improving knee function as compared to corticosteroid injections [64].

Tang et al. conducted a systemic review of 20 RCTs comparing PRP and HA. Patients treated with PRP had substantial improvement in pain in the short term and enhanced functional recovery in the long term as compared to the patients who received HA [65]. Belk et al. performed a systemic review of RCTs comprising 1042 patients with knee OA in their sixth decade of life. Patients administered with PRP and bone marrow aspirate concentration (BMAC) reported better clinical outcomes as compared to patients treated with HA. However, no clinically significant differences were found between PRP and BMAC [66]. Another systemic review of 14 RCTs revealed that L-PRP did not have a remarkable analgesic effect among patients of knee OA as compared to hyaluronic acid. Nonetheless, improvement in stiffness and function was reported at 12 months follow-up [67]. 

Hip and Knee OA

The meta-analysis carried out by Dong et al. reviewed multiple databases and focused on observing outcomes such as the WOMAC, Knee Injury and Osteoarthritis Outcome Score (KOOS), VAS, HHS, and IKDC score. This comprehensive analysis included 24 RCTs, with 21 addressing knee OA and three focusing on hip OA. The results revealed that PRP injections significantly improved WOMAC, VAS, IKDC, and HHS scores compared to other treatments. PRP showed marked benefits in reducing pain and enhancing function, as evidenced by better WOMAC pain, stiffness, and physical function scores. Studies with a low risk of bias consistently reported positive outcomes for PRP, with improvements observed at short-term follow-ups from one to 12 months [68]. However, these findings were contradicted in another systematic review and meta-analysis by Xiong et al. wherein researchers found that there is no statistically significant difference between the experimental and control groups in VAS and WOMAC scores in hip OA. This indicates that PRP injections did not improve the pain in hip OA. However, in the same analysis, it was concluded that the knee, ankle, and temporomandibular joints did have a better outcome in relieving pain with PRP injections [69]. Comparative studies have shown mixed results regarding the superiority of PRP over HA. In a study with 100 patients, both PRP and HA groups exhibited significant improvements in pain and function, but no significant difference was observed between the two treatments at 12 months [70]. Another systematic review including 29 clinical studies reported significant improvements in pain and functional outcomes for PRP-treated patients compared to baseline, with benefits lasting up to 12 months [71]. All RCTs and systemic reviews have been summarized in Tables 1-2.

**Table 1:** Summary of randomized controlled trials of PRP in knee and hip OA PRP: platelet-rich plasma;L-PRP: leukocyte-rich PRP; P-PRP: leukocyte-poor PRP; HA: hyaluronic acid; WOMAC: Western Ontario and McMaster Universities Osteoarthritis Index; VAS: visual analogue scale; CS: corticosteroid; IA: intra-articular; OA: osteoarthritis; PDGF: platelet-derived growth factor; ACS: autologous conditioned serum

Author name (year)	Joint Type	Type of PRP used	No. of injections	Amount per injection	Frequency of injections	Modality compared with	Main Outcomes
Battaglia et al. (2013) [43].	Hip	L-PRP	3	5 ml	Once every week	HA	Significant pain reduction at 4 weeks, not maintained at 16 weeks.
Bennell et al. (2021) [58].	Knee	P-PRP	3	5 ml	Once every week	Saline	Administration of PRP did not result in significant pain relief as compared to saline after 12-month intervals.
Dallari et al. (2016) [42].	Hip	L-PRP	3	5 ml	Once every week	HA and PRP+HA	L-PRP group showed significant clinical improvement with the mean VAS score being the lowest at the 6-month follow-up. The WOMAC scores were better at the 2-month and 6-month follow-ups. Adding L-PRP to HA did not significantly reduce pain.
Di Martino et al. (2022) [60].	Knee	L-PRP vs P-PRP	3	5 ml	Once every week	-	Both types of PRP were equally efficacious in alleviating symptoms at 12 months follow-up.
Di Martino et al. (2019) [61].	Knee	PRP	3	5 ml	Once every week	HA	Functional enhancement and pain relief were reported in both groups. However, neither modality showed superiority over the other.
Di Sante et al. (2016) [44].	Hip	L-PRP	3	5 ml	Once every week	HA	Significant improvement in pain and function at 1 and 3 months. No significant difference at 12 months.
Doria et al. (2017) [41].	Hip	L-PRP	3	5 ml	Once every week	HA	PRP did not perform better in mitigating symptoms than HA at 6-month and 12-month intervals for middle-aged patients with moderate OA.
Elik et al. (2020) [56].	Knee	PRP	3	4 ml	Once every week	Saline	A substantial reduction in WOMAC scores was noted after the six-month follow-up in the PRP group as compared to the placebo. No change in cartilage thickness was observed.
Forogh et al. (2016) [64].	Knee	PRP	1	-	Single Injection	CS	PRP significantly decreased pain and enhanced functional activity as compared to CS.
Huang et al. (2022) [62].	Knee	P-PRP	1	3 ml	Single injection	PRP + Artz vs PRP + HYAJOINT	Similar improvement in VAS and WOMAC pain scores was reported in both IA regimens.
Nouri et al. (2022) [40].	Hip	P-PRP	2	5 ml	Once every 2 weeks	HA and PRP+HA	The improvement in pain and function at the 6-month mark was significantly greater in the PRP + HA and PRP groups compared to the HA group
Partan et al. (2024) [57].	Knee	Multiple PRP	5	2 ml	Once every week	Multiple HA	Marked reduction in pain was reported in both groups, with better outcomes in PRP patients. PDGF-BB levels increased with PRP.
Park et al. (2021) [59].	Knee	L-PRP	1	-	Single injection	HA	PRP showed superior results than HA in improving symptoms.
Pishgahi et al. (2022) [63].	Knee	P-PRP	2	-	Once every week	Dextrose prolotherapy and ACS	Alleviation of pain and betterment in knee function were observed in both the PRP and ACS groups, with superior results in the ACS group. No improvement was reported in dextrose prolotherapy patients.
Raeissadat et al. (2020) [55].	Knee	L-PRP	2	-	4-week interval between 2 doses	Exercise	Significant improvement in WOMAC and VAS scores was reported. Radiological increase in cartilage volume and reduction in synovial inflammation were observed in the PRP group.

**Table 2:** Summary of systemic reviews of PRP in knee and hip OA. PRP: platelet-rich plasma; BMAC: bone marrow aspirate concentration; HA: hyaluronic acid; WOMAC: Western Ontario and McMaster Universities Osteoarthritis Index; VAS: visual analogue scale; P-PRP: leukocyte-poor PRP; L-PRP: leukocyte-rich PRP; IKDC: International Knee Documentation Committee; CS: corticosteroid; OA: osteoarthritis

Author (year)	Joint type	No. of RCTs included	Total no. of patients	Treatment modalities compared to PRP	Conclusions
Belk et al. (2023) [66].	Knee	27	1042	BMAC and HA	PRP and BMAC showed similar efficacy in reducing pain and improving function as opposed to HA. No clinically significant difference was found between PRP and BMAC.
Belk et al. (2022) [48].	Hip	6	211	HA	PRP and HA injections offer similar short-term benefits for hip OA, with no significant differences in improvement across key outcome scores.
Berney et al. (2021) [50].	Hip	5	185	HA and PRP + HA	PRP improved patient outcome scores of WOMAC and VAS at 6 and 12 months, but there was no significant difference compared to patients treated with only hyaluronic acid.
Dong et al. (2021) [68].	Hip and Knee	24	-	Saline (placebo), HA, ozone, and CS	Knee OA patients consistently showed better outcomes as compared to other modalities. PRP does not regularly show improved clinical outcome than HA for hip OA.
Garcia et al. (2020) [49].	Hip	4	334	Placebo, HA and CS	PRP treatment for OA showed improvement in patient-reported outcomes and pain reduction for up to a year but showed no statistically significant difference in pain relief between PRP and HA at short-term, midterm, or long-term follow-ups.
Gazendam et al. (2020) [51].	Hip	11	1353	Saline (placebo), CS, HA and PRP + HA.	Saline injections for hip pain worked equally well as other injectable treatments for both hip pain and function, except HA+PRP, showing significant improvements compared to baseline at 2, 4 and 6 months
Lim et al. (2023) [45].	Hip	8	331	Single vs. multiple doses of PRP < 15 ml and ≥ 15 ml dose of PRP.	PRP reduces pain and improves function in hip OA patients 1 to 2 months after treatment. A single PRP injection works better for pain relief than multiple injections and smaller doses (or P-PRP) are more effective than higher doses (or L-PRP).
Medina-Porqueres et al. (2021) [46].	Hip	4	334	HA	PRP showed varying results for treating hip OA: some studies found it improved pain and function more than HA, especially in the short term, while others saw no clear advantage of PRP over HA in the longer term.
Peng at al. (2022) [67].	Knee	14	1485	HA	LR-PRP showed better WOMAC total, pain, physical function, and IKDC scores at 6 and 12 months compared to HA. No significant difference in adverse events.
Sambe et al. (2023) [47].	Hip	7	478	HA	At six months, PRP treatment led to a significant drop in WOMAC pain scores and some improvement in VAS scores, but there was no notable difference compared to HA at one to two months or 12 months, and both treatments showed similar results for hip dysfunction at six and 12 months.
Tang et al. (2020) [65].	Knee	20	1281	HA	Patients treated with PRP reported greater reduction in pain and better functionality at 6- and 12-month follow-ups as compared with HA.
Xiong et al. (2023) [69].	Hip and Knee	24	1344	Saline (placebo) and HA	PRP significantly reduced pain in knee OA. However, PRP proved ineffective in decreasing pain among patients with hip OA.
Ye et al. (2018) [53].	Hip	4	303	HA	PRP provided significant short-term pain relief but was not superior to HA at 6 and 12 months.
Zaffagnini et al. (2022) [54].	Hip	20	735	Bone marrow adipose tissue and amniotic fluid	All treatment modalities showed potentially beneficial results in mitigating symptoms. However, efficacy decreased as the severity of OA increased.
Zhao et al. (2020) [52].	Hip	11	1060	HA and CS	CS and HA were more effective than the control in reducing pain at 1 and 3 months, with CS outperforming HA at 1 month and being the best short-term treatment for hip OA. PRP, while ranking lower for pain relief at 6 months, still shows promise for short to mid-term effectiveness.

Discussion

The efficacy of PRP in treating hip and knee OA shows promise, particularly in terms of short-term pain relief and functional improvement. PRP can be recommended as a therapeutic option for OA, particularly for patients who do not respond to conventional therapies such as physical therapy, weight management, or other IA injections, and non-surgical treatment options need to be explored. However, the benefits appear to diminish over time, and PRP is not consistently superior to HA. The variability in PRP preparation and administration protocols highlights the need for standardized approaches to optimize outcomes and facilitate comparisons across studies.

The preparation and administration of PRP vary significantly across studies. PRP can be prepared using various methods, including commercial kits and laboratory-based protocols, which can result in significant variability in platelet concentrations, white blood cell content, and growth factor profiles. Variable volumes ranging from 2-6 mL per injection and platelet concentrations 1.7-6 times higher than whole blood are utilized. Some studies used L-PRP, while others used P-PRP. This heterogeneity makes it challenging to compare results across different studies and underscores the need for standardized protocols [72-75]. Interventions fluctuated between different studies from a single injection to multiple injections including twice monthly or three injections administered within two- to three-week intervals. Injections were administered once a week for three consecutive weeks [50, 52].

In several studies, patients did not receive any concurrent treatments such as physical therapy or medications other than standard analgesics. Additionally, many studies had small sample sizes and short follow-up periods, limiting the generalizability of the findings. Di Sante et al. postulated that the lack of a control group and the small sample size were significant limitations in their study. The quality of the studies varies, with some having robust double-blind designs while others show methodological limitations that affect the reliability of their measurements [44, 59, 76]. Another important aspect noted from several studies was that the PRP treatment was more effective on low BMI and younger patient populations. A novel approach could target higher BMI and elderly populations to ensure the safety and efficacy of PRP [44, 45].

PRP injections are generally considered safe, with most studies reporting only mild and self-limiting adverse effects. These include transient pain and swelling at the injection site, which typically resolve within 24-48 hours [51, 67]. No severe adverse events have been reported in the literature reviewed. In a systematic review of 12 RCTs, no significant difference in adverse effects between PRP and HA groups was found, with both treatments showing a low incidence of complications [54]. Another study reported that PRP was associated with fewer complications compared to HA and corticosteroids [77].

Marketing PRP therapies to patients for purposes that lack sufficient evidence is a contentious issue. Several clinics advertise PRP as a "stem cell therapy" for the treatment of diseases including Alzheimer's and Parkinson's, even though there is no evidence to support this claim. Concerns about the potential for patients to be duped and exploited have arisen from the use of direct-to-consumer marketing [78, 79]. Alleging that the new limits would prevent patients from receiving PRP treatment, certain parties have voiced their objection to them. Weak marketing regulations in certain nations allow PRP treatment for various diseases even if there is little evidence to support its use [30]. It is essential to have this regulatory conversation so that we can develop a comprehensive approach that guarantees the efficacy and safety of PRP products while also encouraging innovation and accessibility. Patients, physicians, and researchers have difficulties due to the absence of established procedures. Enforcing uniform laws might make it easier to develop standards for PRP production and labeling, which would reduce the likelihood of unfavorable outcomes and deceptive claims. More precisely framed laws might also provide businesses looking to create PRP therapies a clearer route, which could draw more capital, and hopefully result in more stringent clinical trials [80].

## Conclusions

Novel biological therapeutic techniques are emerging to treat musculoskeletal conditions such as osteoarthritis. PRP is one such modality that has shown potential efficacy for the treatment of osteoarthritis, especially in knee and hip joints. PRP IA injections are particularly recommended in cases of failure of conventional treatment regimens and when non-surgical modalities are necessary.

However, recent clinical trials have shown controversial results regarding the safety and efficacy of PRP in mitigating pain and improving function among patients of knee and hip OA. The lack of uniform preparation techniques, variability in dosage and frequency, and inconsistent composition render these clinical trials debatable. Therefore, it is imperative to establish guidelines regarding standardized preparation and administration and formulate regulations to help clinicians adopt this therapeutic modality in clinical practice.
